# Supplementary material for: Machine learning prediction of emesis and gastrointestinal state in ferrets
Source: PLoS One. 2019 Oct 18;14(10):e0223279. doi: 10.1371/journal.pone.0223279 (PMC6799899; doi:10.1371/journal.pone.0223279)
Supplement: S1 Table — (DOCX) [file pone.0223279.s001.docx]

**S1 Table.** **Summary of feature set for classifier training and testing.**

| Animal | Pre-emetine baseline duration (min) | Early emetine duration (min) | Late emetine duration (min) | # Windows per GI signal | # Features per window | # GI signals with DF |
| --- | --- | --- | --- | --- | --- | --- |
| 14-18 | 0 | 15.6 | 15.6 | 15 | 4 | 3 |
| 16-18 | 6.7 | 14.5 | 14.5 | 6 | 3 | 2 |
| 13-18 | 5.0 | 16.2 | 16.2 | 5 | 3 | 4 |
| 15-18 | 5.2 | 13.2 | 13.2 | 5 | 2 | 2 |
