# Supplementary figures and images for: Machine learning prediction of emesis and gastrointestinal state in ferrets
Source: PLoS One. 2019 Oct 18;14(10):e0223279. doi: 10.1371/journal.pone.0223279 (PMC6799899; doi:10.1371/journal.pone.0223279)

**
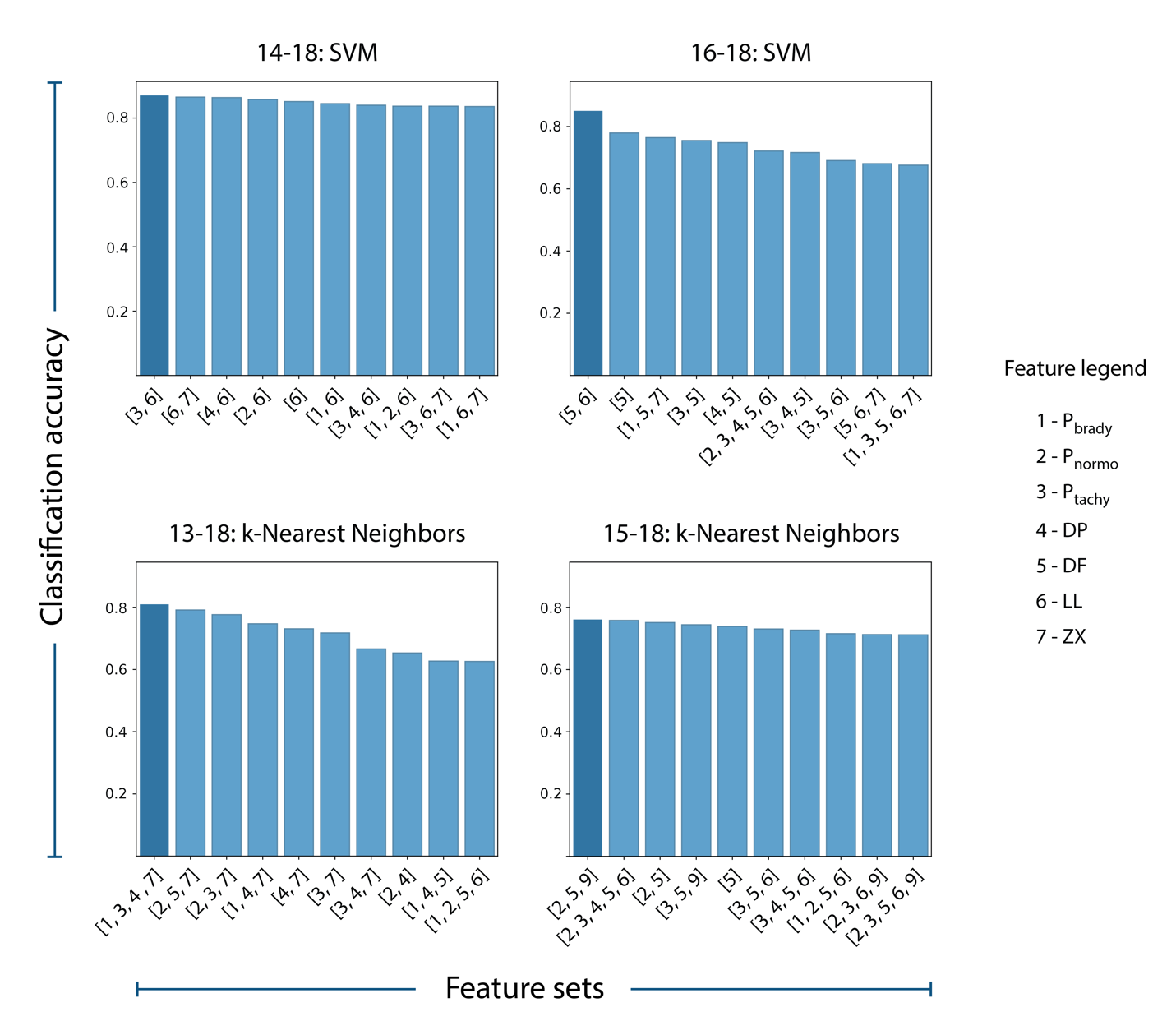
**

**S1 Fig.** Classification accuracy for top 10 feature sets for the optimal algorithm per subject.

Supplement: S1 Fig — (DOCX) [file pone.0223279.s002.docx]
