# Supplementary material for: Machine learning prediction of emesis and gastrointestinal state in ferrets
Source: PLoS One. 2019 Oct 18;14(10):e0223279. doi: 10.1371/journal.pone.0223279 (PMC6799899; doi:10.1371/journal.pone.0223279)
Supplement: S2 Fig — Number of times a particular A) feature and B) gastric segment was observed within the 90th percentile of testing accuracy for k-NN and SVM classifiers after grid search optimization of parameters. (DOCX) [file pone.0223279.s003.docx]

**
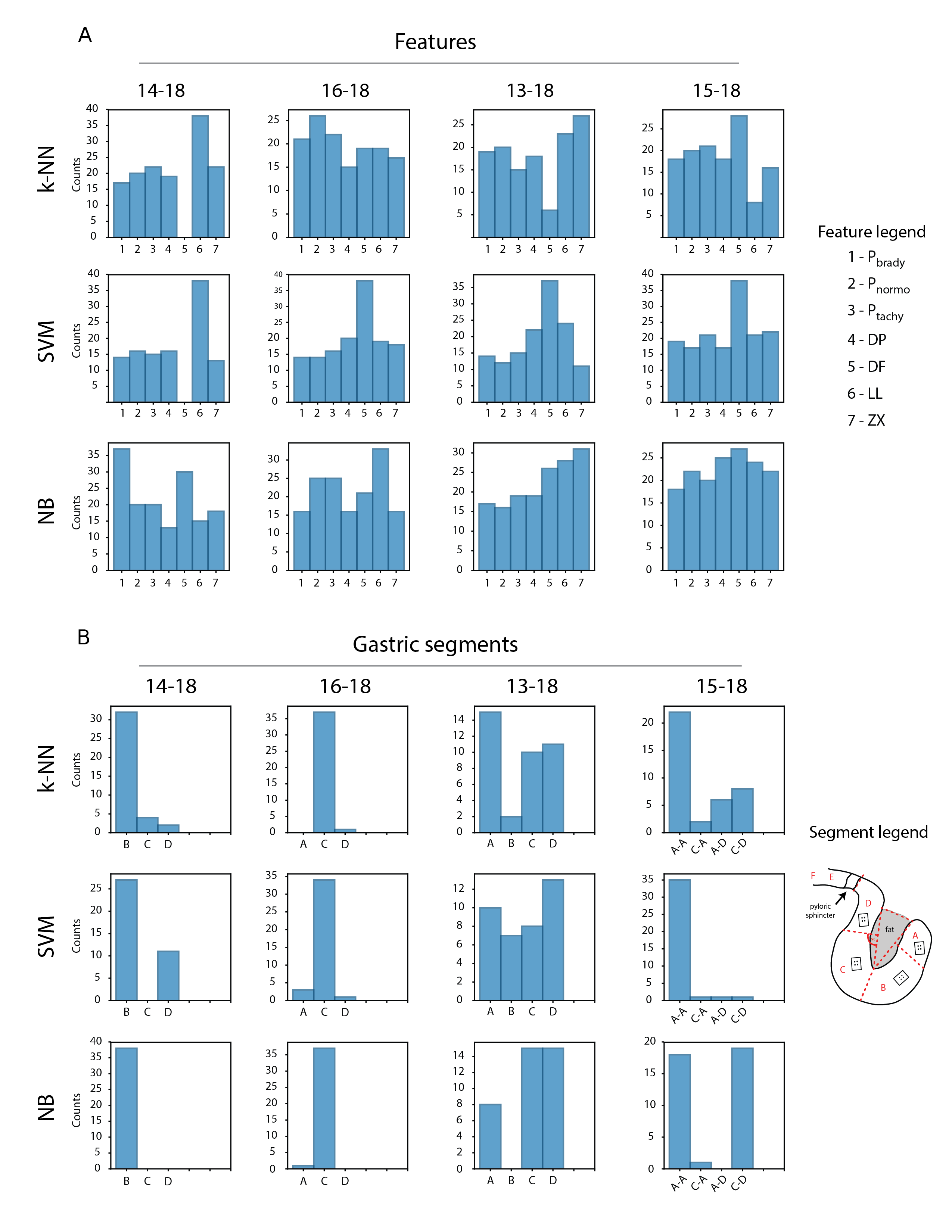
**

**S2 Fig.** Number of times a particular **A)** feature and **B)** gastric segment was observed within the 90^th^ percentile of testing accuracy for k-NN and SVM classifiers after grid search optimization of parameters.
